# Case Report: Resolution of chronic urticaria following treatment of odontogenic infection

**DOI:** 10.12688/f1000research.16836.3

**Published:** 2019-06-06

**Authors:** Susan Tadros, Sameer Bahal, Vasantha Nagendran

**Affiliations:** 1 Immunology Department, Epsom and St Helier NHS Trust, Carshalton, SM5 1AA, UK; 2 Immunology Department, Royal London Hospital, Whitechapel, E1 1BB, UK

**Keywords:** Chronic Spontaneous Urticaria, Dental Infection

## Abstract

**Background:** Chronic spontaneous urticaria (CSU) is a condition characterised by the presence of hives with/without angioedema, that affects individuals on more days than not for 6 weeks or more. The role of infection as a potential trigger for CSU is well described, but the current clinical guidelines do not recommend routine screening for underlying infections.

**Main observations:** We report a case of severe prolonged chronic spontaneous urticaria in a 19-year-old, that went into rapid remission following the treatment of dental infection.

**Conclusions:** Clinicians should recognise the potential role that infection can have in causing chronic urticaria. There should be a low threshold to treat infection in such circumstances.

## Introduction

Urticaria is a dermatological disorder that manifests as raised erythematous lesions that range in size. They are pruritic and typically resolve with no changes to the appearance of the skin. Urticarial lesions may be associated with episodes of swellings known as ‘angioedema’. The role of infection as a potential trigger for urticaria and angioedema is well described but the precise mechanism by which infection induces release of histamine from mast cells is unknown. Infections, including dental infections, have been associated with urticaria; however, current chronic urticaria guidelines do not recommend routine screening for underlying infection ^1, 2^. Instead, they advise targeted investigations, based on the clinical history and examination findings. We report a case of severe chronic spontaneous urticaria that rapidly resolved following root canal treatment.

## Case report

A 19-year-old male patient was referred to the Immunology clinic by his General Practitioner (GP). He presented with a two-month history of urticaria with intermittent episodes of angioedema. His initial symptoms included facial pruritis, periorbital erythema and angioedema involving the upper and lower lips. Within 30 minutes of his first episode of angioedema, he developed widespread urticaria which responded to treatment with antihistamines. The following day, he experienced a recurrence of the symptoms and continued to have almost daily symptoms of urticaria with intermittent episodes of angioedema. He was commenced on an alternative anti-histamine by his GP but continued to develop urticaria and experience swellings of the hands and feet. His treatment was escalated at his initial visit to Immunology Clinic to fexofenadine 180mg twice a day with an additional 10–20mg of cetirizine. In addition, montelukast, a leukotriene receptor antagonist, was commenced.

The number of hives and degree of pruritis were graded using an objective scoring system known as the Urticaria Activity Score 7 (UAS7) that provides a weekly average score out of a maximum score of 42. The patient recorded weekly UAS7 scores of 30, despite treatment with maximum doses of antihistamines and montelukast. Therefore, Anti-IgE therapy with the monoclonal antibody ‘Omalizumab’ was offered. In the interim, he presented to his dentist with a broken tooth and was found to have carious molars requiring root canal treatment. One week after this intervention, his UAS7 score fell to 4 and then to 0, and he has remained in remission (UAS 7 score 0) for 9 months. As he was rather needle-phobic, he was delighted that this obviated the need for Omalizumab injections. Initial investigations including full blood count, renal function, liver function and thyroid function tests were all within the normal ranges.

## Discussion

Chronic spontaneous urticaria (CSU) is defined as daily or almost daily urticaria for at least 6 weeks ^1^. In up to 50% of patients, urticaria may be associated with episodes of angioedema ^3^. These features are the result of degranulation of mast cells with the release of granule contents, predominantly histamine. Patients often present to their GP and are referred for further assessment and management by Immunologists, Allergists or Dermatologists when first line treatment with antihistamines fail to control the symptoms. The mainstay of treatment is high dose antihistamines and leukotriene receptor antagonists ^1^. In recent years, the anti-IgE monoclonal antibody therapy, Omalizumab, has been used as an effective treatment for patients who fail to respond to first and second line therapy.

In cases of CSU, triggers such as food-based allergens or airborne allergens are rarely implicated ^4^. In acute urticaria (defined as having a duration of less than 6 weeks), causes are more likely to be identified. In one study of 79 cases of acute urticaria, 36.7% were secondary to infection ^5^.

A number of studies have demonstrated an increased prevalence of oropharyngeal infections including dental infections, sinusitis and tonsillitis in patients with chronic urticaria ^3, 6, 7^. An early study from 1964 demonstrated radiological evidence of focal dental infection in 29% of their cohort of patients with chronic urticaria ^3^. In addition, cases have been reported of resolution of urticaria after treatment of dental infections ^8, 9^. In one case bacterial cultures from dental lesions grew the gram-negative bacteria *Veillonella parvula* ^10^. It is thought that Lipopolysaccharide from gram negative bacteria induces an inflammatory response characterised by histamine release from mast cells and resulting urticaria ^11^.

Other infections reported to be associated with CSU include *Helicobacter pylori* which is known to have immunomodulatory effects ^12^. However, *H. pylori* eradication in CSU patients has had mixed results ^13– 15^. Infection with viral hepatitis has also been associated with CSU ^16, 17^ and guidelines suggest performing a hepatitis screen if transaminases are abnormal ^1^. However, a systematic review on the subject revealed that the prevalence of hepatitis B and hepatitis C was no greater in CSU patients than compared with the general population ^18^.

The presented case history demonstrates the close temporal relationship between treatment of dental infection and the improvement of urticaria and reduction in medication requirements. Inflammatory markers were not monitored in this case but may have been elevated ^19^. Measurement of markers of the acute inflammatory response, including CRP, can easily be included in assessment of patients with chronic urticaria. Together with a careful history, an elevation in acute inflammatory markers, may highlight the presence of infection/inflammation. Where infection has been excluded, the elevated inflammatory markers may identify patients with more severe chronic urticaria ^20^.

Our patient had failed first and second line treatments for chronic urticaria with persistent and troublesome symptoms. With a UAS 7 >28, demonstrating poorly controlled chronic urticaria, he was eligible to commence anti-IgE therapy ^21^. Monoclonal antibody anti-IgE treatment with Omalizumab is now provided by some immunology and dermatology units in the UK. Patients are given Omalizumab by sub-cutaneous injection once a month for 6 months, and their response is monitored throughout. Although relatively safe, any new treatment is not without the risk of side effects. In addition, the treatment is costly, and should be reserved for patients who have severe CSU that fail to respond to treatment with the maximum dose of anti-histamine treatment and leukotriene receptor antagonists.

Our case history illustrates the importance of searching for infections, including odontogenic infections, prior to commencing immunosuppression or anti-IgE therapy in patients who are resistant to first line treatment of CSU.

## Consent

Written informed consent was obtained from the patient for the publication of their clinical details.

## Data availability

No data is associated with this article.
