# Supplementary material for: Case Report: Resolution of chronic urticaria following treatment of odontogenic infection
Source: F1000Res. 2019 Jun 6;7:1738. Originally published 2018 Nov 2. [Version 3] doi: 10.12688/f1000research.16836.3 (PMC6545821; doi:10.12688/f1000research.16836.3)
Supplement: Peer review history

# Peer review: Reviewer response for version 3

**DOI:** 10.5256/f1000research.21444.r49625

**Published:** 2019-06-10

**Authors:** Riccardo Asero

**Affiliations:** 1 Ambulatorio di Allergologia, Clinica San Carlo, Paderno Dugnano (Milan), Italy

The authors have responded to my comments.

I have read this submission. I believe that I have an appropriate level of expertise to confirm that it is of an acceptable scientific standard.

# Peer review: Reviewer response for version 2

**DOI:** 10.5256/f1000research.21100.r49180

**Published:** 2019-05-31

**Authors:** Riccardo Asero

**Affiliations:** 1 Ambulatorio di Allergologia, Clinica San Carlo, Paderno Dugnano (Milan), Italy

Tadros and co-workers report a case of severe chronic urticaria unresponsive to second generation antihistamines given at higher than licensed doses that resolved following the treatment of dental infection.

 General comments

 The clinical case is nicely described and easy to follow.

 Specific points

 The authors completely overlook the last version of the EAACI/GA2LEN/EDF/WAO guidelines ^1^ that address most of the points discussed in the present article.

 The role of infection of CSU has long been debated (as also shown by the fact that many of the cited references are dated) but an unquestionable proof is missing. Those who, like this reviewer, have been dealing with urticaria patients for decades can easily remember that many years ago patients underwent systematically dental X-rays followed by root canal treatment (when necessary), in most cases without any benefit on the skin disease. The same holds true for eradication of *H. pylori* infection. The EAACI guidelines include the research for infectious disease within the “extended diagnostic program”, based on clinical history.

 CRP is included on the routine diagnostic tests to be performed in all CSU patients. The last part of the discussion addresses Omalizumab, but this is out of scope of the present case report as this drug was eventually not given.

 Minor points

 The maximum theoretical score of UAS 7 is 42 (6 x 7) and not 40.

I have read this submission. I believe that I have an appropriate level of expertise to confirm that it is of an acceptable scientific standard, however I have significant reservations, as outlined above.

## References

[1] ZuberbierTAbererWAseroRAbdul LatiffAHBakerDBallmer-WeberBBernsteinJABindslev-JensenCBrzozaZBuense BedrikowRCanonicaGWChurchMKCraigTDanilychevaIVDresslerCEnsinaLFGiménez-ArnauAGodseKGonçaloMGrattanCHebertJHideMKaplanAKappAKatelarisCHKocatürkEKulthananKLarenas-LinnemannDLeslieTAMagerlMMathelier-FusadePMeshkovaRYMetzMNastANettisEOude-ElberinkHRosumeckSSainiSSSánchez-BorgesMSchmid-GrendelmeierPStaubachPSussmanGToubiEVenaGAVestergaardCWediBWernerRNZhaoZMaurerMEndorsed by the following societies: AAAAI, AAD, AAIITO, ACAAI, AEDV, APAAACI, ASBAI, ASCIA, BAD, BSACI, CDA, CMICA, CSACI, DDG, DDS, DGAKI, DSA, DST, EAACI, EIAS, EDF, EMBRN, ESCD, GA²LEN, IAACI, IADVL, JDA, NVvA, MSAI, ÖGDV, PSA, RAACI, SBD, SFD, SGAI, SGDV, SIAAIC, SIDeMaST, SPDV, TSD, UNBB, UNEV and WAO: The EAACI/GA²LEN/EDF/WAO guideline for the definition, classification, diagnosis and management of urticaria. *Allergy*.73(7) : 10.1111/all.13397 1393-1414 10.1111/all.13397 29336054

# Peer review: Reviewer response for version 2

**DOI:** 10.5256/f1000research.21100.r40188

**Published:** 2019-05-31

**Authors:** Patrick Yong

**Affiliations:** 1 Department of Allergy and Clinical Immunology, Royal Surrey County Hospital, Guildford, UK

This report presents a case of CSU which improved following treatment of dental infection. This is a recognised association with CSU, and serves as a useful reminder to clinicians treating patients with CSU.

 The case report and summary of the literature is concise and to the point. The only suggestion I would make is that it may be worth citing other urticaria guidelines (e.g. EAACI 2018 which is more recent), and making it clearer in the introduction that infection (along with various other causes) is listed as a cause of CSU in guidelines. Extended testing for the various causes is guided by the history. Also, some of the initial tests in the EAACI guidelines i.e. FBC/CRP can give a hint that there may be infection.

 One small correction in the case report is that the UAS7 score has a maximum of 42, not 40.

I have read this submission. I believe that I have an appropriate level of expertise to confirm that it is of an acceptable scientific standard.

# Peer review: Reviewer response for version 2

**DOI:** 10.5256/f1000research.21100.r48539

**Published:** 2019-05-15

**Authors:** Sinisa Savic

**Affiliations:** 1 Section of Musculoskeletal Disease, Leeds Institute of Molecular Medicine, NIHR Leeds Musculoskeletal Biomedical Research Unit, Chapel Allerton Hospital, University of Leeds, Leeds, UK

The authors have adequately addressed all my comments.

I have read this submission. I believe that I have an appropriate level of expertise to confirm that it is of an acceptable scientific standard.

# Peer review: Reviewer response for version 1

**DOI:** 10.5256/f1000research.18406.r45040

**Published:** 2019-04-01

**Authors:** Sinisa Savic

**Affiliations:** 1 Section of Musculoskeletal Disease, Leeds Institute of Molecular Medicine, NIHR Leeds Musculoskeletal Biomedical Research Unit, Chapel Allerton Hospital, University of Leeds, Leeds, UK

This case report describes a patient who developed CSU refractory to 1 ^st ^and 2 ^nd ^line therapies. His CSU resolved promptly after treatment of the concurrent dental infection. 

 This case illustrates importance of searching for underlying causes of CSU as part of routine clinical assessment. The authors state that routine infection screen is not recommended by the current guidelines, however the guidelines state: ‘The diagnosis is based primarily on the clinical history. Investigations are determined by the clinical history and presentation, but may not be necessary”. Although infection screen is not specifically mentioned, taking a thorough clinical history as suggested by the guidelines should include enquiry about possible infections and subsequent investigations might include an infection screen. 

 One of the reasons why infections might be overlooked when assessing CSU, is that the past recommendations for routine screening for H. *Pylori *and its eradication failed to produce desired outcomes ^1^. There are other examples in the literature where chronic infection has been linked with CSU, and one such example is HepC and HepB. However, a comprehensive review of the literature failed to find an obvious associations, and routine screening is not recommended ^2^. 

 In summary, this is well written article with a useful message, but discussion regarding the role of infection triggers in CSU should be more comprehensive and include the points mentioned above.

I have read this submission. I believe that I have an appropriate level of expertise to confirm that it is of an acceptable scientific standard, however I have significant reservations, as outlined above.

## References

[1] CurthHMDinterJNigemeierKKüttingFHunzelmannNSteffenHM: Effects of Helicobacter pylori Eradication in Chronic Spontaneous Urticaria: Results from a Retrospective Cohort Study. *Am J Clin Dermatol*.2015;16(6) : 10.1007/s40257-015-0152-6 553-8 10.1007/s40257-015-0152-6 26334425

[2] KolkhirPPereverzinaNOlisovaOMaurerM: Comorbidity of viral hepatitis and chronic spontaneous urticaria: A systematic review. *Allergy*.2018;73(10) : 10.1111/all.13482 1946-1953 10.1111/all.13482 29786879
